# Defining phenotypes of long-term lithium and valproate response, including combination therapy: a modified application of the Alda scale in patients with bipolar disorders

**DOI:** 10.1186/s40345-020-00199-w

**Published:** 2020-11-20

**Authors:** Jinyoung Lee, Ji Hyun Baek, Dongbin Lee, Sung Woo Ahn, So-Yung Yang, Yujin Choi, Yong Chun Bahk, Kyung Sue Hong

**Affiliations:** 1 Department of Psychiatry, Sungkyunkwan University School of Medicine, Samsung Medical Center, Seoul, Republic of Korea; 2 Present Address: Department of Psychiatry, National Health Insurance Service, Ilsan Hospital, Goyang-si, Republic of Korea; 3 Center for Clinical Research, Samsung Biomedical Research Institute, Seoul, Republic of Korea

**Keywords:** Bipolar disorder, Long-term treatment response, Lithium, Valproate, Alda scale, Clinical correlates

## Abstract

**Background:**

When evaluating the long-term treatment response to mood stabilizers using the Alda scale, mood stabilizer combination therapy is typically considered a confounding factor, and patients receiving combination therapy are excluded from the analysis. However, this may result in bias if those under combination therapy are worse treatment responders. This study aims to explore whether the Alda scale is applicable to patients taking lithium and valproate combination therapy. We compared long-term treatment response in patients receiving monotherapy and combination therapy of the two drugs, and investigated clinical correlates of the responses to each drug.

**Methods:**

The study subjects consisted of 102 patients with bipolar I (BD-I) or bipolar II (BD-II) disorder who had been undergoing maintenance treatment with lithium and/or valproate for more than 2 years at a single specialized bipolar disorder clinic. Long-term treatment response was measured using the Alda scale and compared among the lithium monotherapy group, the valproate monotherapy group, and the mood stabilizer combination group. Clinical correlates of long-term treatment response were evaluated in lithium users and valproate users separately.

**Results:**

There were no significant differences in terms of baseline illness characteristics among groups. The combination group showed the worst treatment response for all the response measurements applied. This group also had the higher rate of ‘poor responder’ with a statistically significant difference compared to valproate group. Older age at onset and (hypo)manic episode at onset showed significant positive associations with total Alda score in lithium users, while comorbid anxiety disorders, obsessive–compulsive disorder and mixed episode showed significant negative associations in valproate users.

**Conclusions:**

The combination group had poorer long-term treatment response but did not show distinct clinical characteristics compared to the monotherapy groups. When exploring the long-term effects of mood stabilizers, excluding patients undergoing combination treatment could result in bias because they may represent a poor response group. The long-term treatment responses of lithium and valproate had different clinical correlates.

## Background

Almost all patients with bipolar disorder (BD) require maintenance pharmacotherapy to prevent subsequent episodes, complications, and residual disabilities even after acute episodes (Grunze et al. 2018; Yatham et al. 2018). Lithium and valproate are commonly used for maintenance treatment of BD, and several newer drugs have been added to the list of first-line recommendations (Miura et al. 2014; Goodwin et al. 2016; Yatham et al. 2018). However, the clinical and biological correlates of long-term treatment response to individual mood stabilizers are not well-defined (Pisanu et al. 2018).

In the last decade, the feasibility of large-scale pharmacogenomic studies has rapidly increased, and the international Consortium on Lithium Genetics (ConLiGen) and other groups have tried to identify the genetic basis of responsiveness to mood stabilizers (Manchia et al. 2013; Rybakowski 2013; Hou et al. 2016; Zhu et al. 2017). Considering the recurrent and biphasic course of BD, exploration of the genetic and clinical factors associated with maintenance therapy requires long-term observation. Therefore, retrospective measurement in naturalistic clinical settings is inevitable. In addition, the need to incorporate clinical correlates into pharmacogenetic analyses has been recognized (Lin et al. 2018).

The most widely adopted measurement tool for long-term treatment response to mood stabilizers in BD is the Alda scale (Manchia et al. 2013). It retrospectively measures global treatment outcome (A score) and several confounding factors that affect treatment outcome (B score). The total score is a composite score acquired by subtracting B from A (Grof et al. 2002). Recently, it has been suggested that the Alda scale requires modification. Scott et al. (Scott et al. 2017, 2019) proposed a validity issue regarding use of a single scoring system for the B score for multiple heterogenous aspects of confounders. They suggested a machine-learning approch to overcome this problem. Other researchers used global outcome scores (A score) in subjects with comparable conditions in terms of confounding factors, i.e., among low B scorers (Lee et al. 2011; Chen et al. 2016; Hou et al. 2016). In addition, whether the effects of the numbers and frequencies of previous episodes before starting the index medication (reflected in B1 and B2) need to be controlled as confounding factors or included as target candidates of outcome predictors depends on the study purpose and design. If the study aims to explore predictors or correlates of drug response, the previous course needs to be included in the analysis as a main independent variable. In that case, the response measurement system of the Alda scale needs to be modified.

Another important issue to be considered when assessing the long-term response to mood stabilizers is the enrollment of patients who are treated with mood stabilizer combination therapy. Until recently, most pharmacological studies measuring long-term treatment response to specific mood stabilizers recruited patients undergoing monotherapy (Gyulai et al. 2003; Pfennig et al. 2010; Manchia et al. 2013; Sportiche et al. 2017). In clinical practice, however, concomitant use of more than one mood stabilizer happens frequently, especially in patients with poor treatment response (Baek et al. 2014). Therefore, excluding those patients might generate a skewed distribution of subjects in terms of drug response, which results in a decrease in statistical power in clinical and biomarker studies. In a previous study by some of the present authors (Ahn et al. 2017), we tried to identify clinical correlates of long-term mood stabilizer response in patients taking lithium and/or valproate. In that study, we used the Alda scale and treated the lithium and valproate combination as a single index medication, and identified overall response correlates for the two drugs.

This study aimed to compare long-term treatment response among groups of patients treated with lithium and valproate. In measuring treatment response using the Alda scale, we included patients receiving mood stabilizer combination therapy and intended to explore whether the Alda scale is applicable in subjects under mood stabilizer combination therapy. We also explored the clinical correlates of the response to each drug.

## Methods

### Subjects

Patients who met DSM-IV criteria for bipolar I (BD-I) or bipolar II (BD-II) disorder and who had received treatment with lithium and/or valproate for more than 2 years between March 2009 and September 2017 at the Bipolar Disorder Clinic of Samsung Medical Center, a tertiary-care university-affiliated hospital, were included in the study. Two drugs, lithium and valproate, were the index medications for the study, and users of both drugs were included in the sample population. The lithium and valproate combination group included patients who had received combination therapy for more than 2 years. The best treatment was provided to each patient based on treatment guidelines (Grunze et al. 2013; Yatham et al. 2018), clinicians’ experience, and patients’ special concerns regarding adverse effects of the drug. We limited the age range of the subjects to between 18 and 55 years because older patients with bipolar disorder could have distinct clinical courses and treatment responses (Sajatovic et al. 2015). Those who showed evidence of neurologic disorders or general medical conditions related to mental symptoms were excluded. A total of 102 patients who met the above criteria and agreed to participate were enrolled in the study. All subjects had previously participated in the authors’ clinical and genetic studies (Baek et al. 2011, 2016, 2019; Yang et al. 2015; Ahn et al. 2017), and 80 of them were involved in the previous study on long-term response to mood stabilizers (Ahn et al. 2017). This study was approved by the Institutional Review Board of Samsung Medical Center.

### Assessment of clinical characteristics

Comprehensive disease characteristics had been evaluated in previous studies described elsewhere (Baek et al. 2011, 2016, 2019; Yang et al. 2015; Ahn et al. 2017). Each evaluation was carried out by a direct interview using the revised version of the Korean version of the Diagnostic Interview for Genetic Studies (Joo et al. 2004) or the Structured Clinical Interview for DSM-IV Axis I Disorders (SCID). Basic demographic and clinical characteristics, clinical course of BD, and manifested symptoms of manic and depressive episodes on a lifetime basis were evaluated (Table 1).

**Table 1:** Comparison of baseline characteristics between medication groups

Demographic and illness characteristics	Lithium, (n = 29)^1^	Valproate (n = 56)^2^	Combination, (n = 17)^3^	Group difference	
				Statistic	p-value
Sex [male, n (%)]	11 (37.9)	17 (30.4)	7 (41.2)	χ^2^ = 0.913	0.686
Age (current) [years, mean (SD)]	34.2 (9.2)	31.6 (8.65)	30.6 (8.3)	F = 1.113	0.332
Education [years, mean (SD)]	15.3 (2.3)	15.0 (2.0)	14.6 (1.7)	F = 0.617	0.542
Diagnosis [BD-I, n (%)]	28 (96.6)	44 (78.6)	15 (88.2)	FE	0.063
Age at onset [years, mean (SD)]	24.9 (9.3)	22.0 (7.1)	23.8 (8.4)	F = 1.362	0.261
Polarity at first episode [(hypo) manic, n (%)]	15 (51.7)	23 (41.1)	11 (48.0)	χ^2^ = 3.139	0.208
Pre-index-medication illness duration [years, mean (SD)]^a^	7.8 (5.8)	8.3 (6.6)	7.1 (4.5)	F = 0.239	0.788
Frequency of episodes [frequent, n (%)]					
Total episodes	6 (20.7)	14 (25.0)	4 (23.5)	χ^2^ = 0.197	0.906
Major depressive episodes	5 (17.2)	10 (17.9)	4 (23.5)	χ^2^ = 0.328	0.849
(Hypo)manic episodes	6 (20.7)	15 (26.8)	5 (29.4)	χ^2^ = 0.539	0.764
Family history of psychiatric disorders [present, n (%)]	18 (62.1)	34 (60.7)	9 (61.0)	χ^2^ = 0.414	0.813
Family history of mood disorders [present, n (%)]	12 (41.4)	21 (37.5)	6 (35.3)	χ^2^ = 0.196	0.906
Any anxiety disorder [present, n (%)]	5 (17.2)	13 (23.2)	1 (5.9)	χ^2^ = 2.636	0.268
Obsessive–compulsive disorder [present, n (%)]	5 (17.2)	10 (17.9)	1 (5.9)	FE	0.465
Seasonality [present, n (%)]	4 (13.8)	21 (21.4)	4 (23.5)	χ^2^ = 1.541	0.819
Rapid cycling [present, n (%)]	1 (3.4)	3 (5.4)	0 (0.0)	FE	1.000
Psychotic symptoms [present, n (%)]	26 (89.7)	41 (73.2)	13 (76.5)	χ^2^ = 3.099	0.212
Mixed episodes	8 (27.6)	13 (23.6)	3 (17.6)	χ^2^ = 0.585	0.746

Symptoms of (hypo)manic episodes	Present, n (%)	Present, n (%)	Present, n (%)	χ^2^	p-value
Elevated mood	25 (86.2)	39 (69.6)	14 (82.4)	3.306	0.192
Irritability	12 (41.4)	24 (42.9)	8 (47.1)	0.145	0.930
Grandiosity	23 (79.3)	36 (64.3)	11 (64.7)	2.149	0.341
Decreased sleep need	27 (93.1)	41 (73.2)	16 (94.1)	7.143	0.028^b^
Talkativeness	23 (79.3)	47 (83.9)	13 (76.5)	0.592	0.744
Flight of idea	24 (82.8)	39 (70.9)	12 (70.6)	1.539	0.463
Distractibility	27 (93.1)	44 (78.6)	15 (88.2)	FE	0.186
Hyperactivity	27 (93.1)	49 (87.5)	16 (94.1)	FE	0.732
Excessive involvement in activity	22 (75.9)	36 (64.3)	71 (69.6)	1.664	0.435
Delusion	20 (69.0)	28 (50.0)	12 (70.6)	4.003	0.135
Hallucination	6 (20.7)	16 (29.1)	6 (35.3)	1.254	0.534

Symptoms of depressive episodes	Present, n (%)	Present, n (%)	Present, n (%)	χ^2^	p-value
Depressed mood	24 (82.8)	45 (80.4)	10 (13.2)	4.116	0.128
Decreased interest	23 (79.3)	42 (75.0)	10 (58.8)	2.449	0.294
Appetite change					
No appetite change	13 (44.8)	25 (44.6)	9 (52.9)	0.387	0.824
Decreased appetite	12 (41.4)	21 (37.5)	5 (29.4)	0.660	0.719
Increased appetite	2 (6.9)	5 (8.9)	1 (5.9)	FE	1.000
Insomnia	19 (65.5)	40 (71.4)	7 (41.2)	5.238	0.073
Hypersomnia	8 (27.6)	16 (28.6)	3 (17.6)	0.826	0.662
Agitation	8 (27.6)	15 (26.8)	5 (29.4)	0.046	0.977
Retardation	12 (41.4)	22 (39.3)	3 (17.6)	3.098	0.212
Fatigue or loss of energy	23 (79.3)	40 (71.4)	10 (58.8)	2.212	0.331
Guilty feeling or worthlessness	17 (58.6)	28 (50.0)	50 (49.0)	3.707	0.157
Indecisiveness (concentration)	23 (79.3)	41 (73.2)	10 (58.8)	2.286	0.319
Suicidal ideation	15 (51.7)	31 (55.4)	8 (47.1)	0.385	0.825
Delusion	8 (27.6)	15 (26.8)	6 (35.3)	0.478	0.787
Hallucination	3 (10.3)	9 (16.1)	2 (11.8)	FE	0.923

SD, standard deviation; BP-I, bipolar I disorder; FE, Fisher’s exact test

^a^Duration from the illness onset to starting the index medications

^b^Significant difference in lithium vs. valproate group: p = 0.033; in valproate vs. combination group: p = 0.047; post-hoc analysis was conducted using Bonferroni’s method

^1^Lithium monotherapy group

^2^Valproate monotherapy group

^3^Lithium and valproate combination group

Frequent experience of episodes was defined as having mood episodes, either depressive or (hypo)manic episodes, more than once per year after illness onset; we defined experience of the frequent episode based on frequency of the episode > 25%ile of the total patient sample calculated from our previous study with larger sample sizes (Baek et al. 2019). The polarity of onset was defined as the type of the first episode, where 0 denoted depressive and 1 denoted (hypo)manic. Rapid cycling and mixed episodes were defined following the DSM-IV-TR criteria. We measured seasonality using the Seasonal Pattern Assessment Questionnaire (SPAQ) (Rosenthal et al. 1987). We defined seasonality as having SPAQ-defined subsyndromal or syndromal seasonal affective disorder. Psychotic symptoms were defined as the experience of psychotic symptoms at least once in the patient’s lifetime. Details of symptoms during the most severe (hypo)manic or depressive episode were evaluated using the definition of each symptom criterion in the DSM (Association, AP 2013).

### Assessment of treatment response

Long-term response to treatment was evaluated through retrospective reviews of clinical records. When possible, additional information was obtained directly from the patients during their visits to the outpatient department. Assessments were performed using the Alda scale (Grof et al. 2002) and the overall section of item III of the CGI-BP (CGI-BP-III-O) (Spearing et al. 1997) at the same time. Treatment response in the combination group was evaluated based on the period when the patients received combination therapy.

The Alda scale consists of two rating sections. The Alda A score measures the degree of improvement during the course of treatment, from 0 to 10. The Alda B score evaluates confounding factors that affect the outcome independently of the medication (Criterion B) (Grof et al. 2002). The B1 and B2 parameters measure the number and frequency of previous episodes, while B3 and B4 measure whether subjects received medication for a sufficient period of time and were adequately compliant. B5 assesses the usage of additional medications to improve symptoms. The total Alda score ranges from 0 to 10 and is obtained by subtracting score B from score A. A higher Alda score indicates a better response to treatment. Additionally, we classified participants into two response groups (‘good/moderate responders’ vs. ‘poor responders’). The best-fit theoretical model of two component with the cut-off point at a total score of 4.5 was set by frequentist mixture analysis in our previous study (Ahn et al. 2017). Therefore, a total score 5 or higher was defined as a good/moderate response and a score of 4 or lower was defined as a poor response.

The CGI-BP-III-O measures the degree of symptom improvement from the worst phase of illness prior to the initiation of the index medication; it ranges from 1 (very much improved) to 4 (no change) to 7 (very much worse).

Two research psychiatrists and the clinician who treated each patient (KSH, JHB, SWA, S-YY, and JL) independently reviewed the hospital records and had meetings to determine the consensus score on the treatment response.

### Statistical analysis

We divided the subjects into three groups, the lithium monotherapy group (lithium group), the valproate monotherapy group (valproate group), and the lithium and valproate combination group (combination group), in order to compare participants’ baseline characteristics and long-term treatment response. Comparisons of demographic and clinical variables and treatment response between the three medication groups were performed using the chi-square test (or Fisher’s exact test) for categorical variables and analysis of variance (ANOVA) for continuous variables. Post-hoc analyses were done using the Bonferroni method for categorical variables and Tukey’s test for continuous variables. The Pearson correlation coefficient was calculated to measure the correlation between the scores of treatment response measures.

We conducted simple regression analyses to explore the clinical correlates of the long-term treatment response to each drug. We performed these analyses excluding the combination group. The Alda total score was entered as a dependent variable, and each clinical factor was separately entered as an independent variable.

Probability (p) values less than 0.05 were considered statistically significant. All statistical analyses were done with IBM SPSS version 25.0.

## Results

### Baseline characteristics of the study subjects

Among all 102 subjects, 29 (28.4%) received lithium, 56 (54.9%) received valproate, and 17 (16.7%) received both lithium and valproate (combination group). Eighty-seven (85.3%) had BD-I and fifteen (14.7%) had BD-II. The mean duration of medication was 95.4 months (standard deviation: 70.9, range: 24–192). All subjects had received the index medication for two or more years (B3 = 0), and most of them showed adequate compliance, i.e., B4 = 0 or 1 in 90.2% of the subjects. Table 1 shows the detailed baseline characteristics of the participants. No significant differences were observed among the groups in terms of sociodemographic and disease characteristics including psychiatric comorbid conditions, seasonality, presence of psychotic symptoms, and mixed episodes. Of the manic symptoms measured, decreased need for sleep was more frequently observed in the valproate group compared to the lithium group (post-hoc p = 0.033) and the combination group (post-hoc p = 0.047). No significant differences were observed among groups regarding depressive symptoms.

### Comparison of treatment response among the three medication groups

Significant differences of treatment response were observed among the three medication groups for all the response measurements, and the combination group showed the worst response (Table 2).

**Table 2:** Comparison of treatment responses between medication groups

	Lithium, (n = 29)^1^	Valproate, (n = 56)^2^	Combination, (n = 17)^3^	Statistic	*p*	Post-hoc analysis^a^		
						^1^vs.^2^	^1^vs.^3^	^2^vs.^3^
Patients total (N = 102)								
Alda A [mean (SD)]	6.4 (2.0)	7.1 (1.7)	5.5 (1.4)	F = 6.631	0.002	0.134	0.200	0.002
Alda C [mean (SD)]	2.9 (2.4)	4.1 (2.3)	1.5 (1.7)	F = 9.736	< 0.001	0.038	0.133	< 0.001
Poor responder [n (%)]	21 (72.4)	34 (60.7)	16 (94.1)	FE	0.022	0.495	0.260	0.023
CGI-BP-III-O [mean (SD)]	2.1 (1.0)	2.0 (0.9)	2.9 (1.0)	F = 5.816	0.004	0.919	0.019	0.003

	Lithium, (n = 28)^1^	Valproate, (n = 44)^2^	Combination, (n = 15)^3^	Statistic	*p*	Post-hoc analysis^a^		
						^1^vs.^2^	^1^vs.^3^	^2^vs.^3^
Bipolar I disorder (N = 87)								
Alda A [mean (SD)]	6.4 (2.0)	7.2 (1.4)	5.5 (1.9)	F = 5.822	0.004	0.139	0.246	0.004
Alda C [mean (SD)]	2.8 (2.4)	4.1 (2.3)	1.6 (1.8)	F = 7.799	0.001	0.047	0.228	0.001
Poor responder [n (%)]	21 (75.0)	26 (59.1)	14 (93.3)	FE	0.034	0.310	0.410	0.033
CGI-BP-III-O [mean (SD)]	2.0 (0.7)	1.7 (0.7)	2.2 (0.6)	F = 3.042	0.053	–	–	–

SD, standard deviation; Alda A, A score of the Alda scale (Grof et al. 2002); Alda C, total score of the Alda scale (Grof et al. 2002); CGI-BP-III-O, item III of the Clinical Global Impressions scale for use in bipolar illness, overall (Spearing et al. 1997); FE, Fisher’s exact test

^a^Post-hoc analysis was conducted using Tukey’s test

^1^Lithium monotherapy group

^2^Valproate monotherapy group

^3^Lithium and valproate combination group

The combination group showed a greater CGI-BP-III-O score compared to the lithium group (post-hoc p = 0.019), and the valproate group (post-hoc p = 0.003). In addition, the combination group showed a lower Alda A score (post-hoc p = 0.002) and total score (post-hoc p < 0.001) in comparison with the valproate group. The valproate group showed a greater total Alda score compared to the lithium group (post-hoc p = 0.038). When classifying patients into two groups depending on the cut-off score set from our previous study (good/moderate responder vs. poor responder), the combination group had the highest rate of ‘poor responder’ with a statistically significant difference compared to the valproate group (post-hoc p = 0.023).

The three treatment outcome measures were highly correlated with one another (Additional file 1: Table S1).

**Fig. 1:**
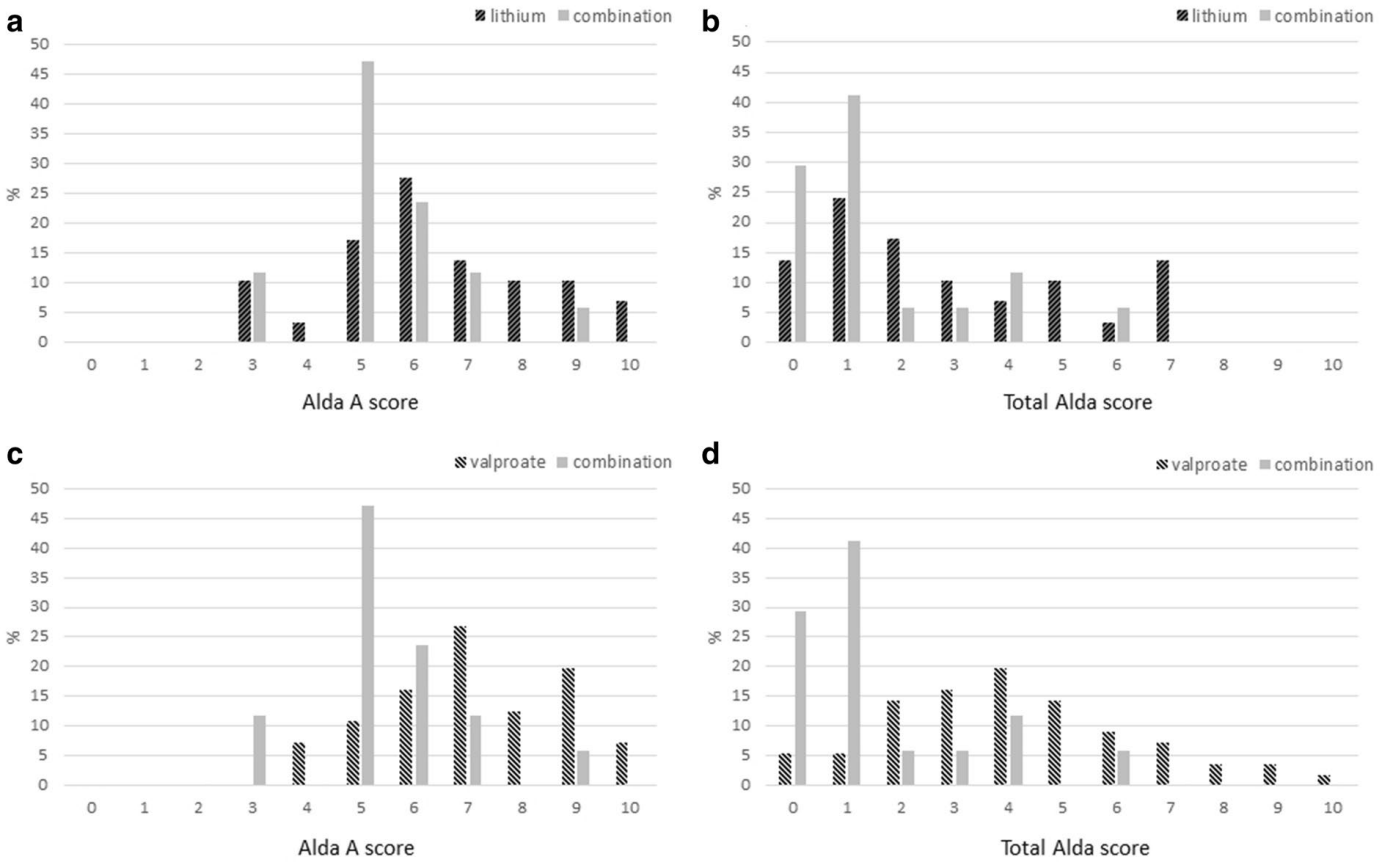
Distribution of Alda scale scores in the monotherapy and combination groups. The x-axis illustrates the scores of the Alda and the y-axis illustrates percentage of patients in each group. **a** Alda A scores of the lithium users. **b** Total Alda scores of lithium users. **c** Alda A scores of valproate users. **d** Total Alda scores of valproate users

Figure 1 illustrates the comparative distributions of the Alda scale scores between the combination group and the monotherapy groups. The combination group generally had lower Alda A and total scores compared to the monotherapy groups.

We additionally compared treatment response in the subset of subjects with BD-I (n = 87). These analyses showed similar results to those found in all subjects (Table 2). However, no significant difference was detected in terms of CGI-BP-III-O score.

### Previous treatments of the combination group

We reviewed details of previous treatment histories of the combination group (Table 3). We explored if treatment regimens recommended for maintenance therapy in the CANMAT-ISBD guideline (Yatham et al. 2018) was administered on each patient. In three patients (patient number 1, 3, and 9 in Table 3), lithium and valproate combination therapy was tried as the second strategy (3/17 = 17.6%). In others, it was tried as the third or later treatment strategy (14/17 = 82.4%). Valproate and atypical antipsychotics combination therapy was the most commonly applied strategy (13/17 = 76.5%) before the current medications, followed by lithium and atypical antipsychotics combination therapy (10/17 = 58.8%). Antidepressant was tried in only one patient to alleviate obsessive compulsive symptoms (patient number 14 in Table 3).

**Table 3:** Summary of previous and current medications for the maintenance therapy in the combination group

Case	Previous medication						Combination of LiVal	
	Lith^1^	Val^2^	Lamo^3^	AAP^4^	Lith + AAP^5^	Val + AAP^6^	Current medication	Order^a^
1	X	X	X	X	X	O	LiVal + AAP	Val → LiVal
2	X	X	X	O	X	O	LiVal + AAP	Val → LiVal
3	X	X	X	X	X	O	LiVal + AAP	Val → LiVal
4	X	X	X	O	X	O	LiVal + AAP	Val → LiVal
5	X	X	X	O	O	X	LiVal + AAP	Lith → LiVal
6	X	X	O	X	O	O	LiVal + AAP	Val → Lith → LiVal
7	X	X	X	O	O	X	LiVal + AAP	Lith → LiVal
8	X	X	X	X	O	O	LiVal + AAP	Lith → Val → LiVal
9	X	O	X	X	X	X	LiVal	Val → LiVal
10	X	X	X	X	O	O	LiVal + AAP	Lith → Val → LiVal
11	X	X	X	O	O	O	LiVal + AAP	Lith → Val → LiVal
12	O	O	X	O	O	O	LiVal + AAP	Val → Lival → Lith → LiVal
13	X	O	O	X	O	O	LiVal + AAP	Val → LiVal → Val → LiVal
14	X	O	X	O	X	O	LiVal + AAP	Val → LiVal
15	X	X	X	O	O	O	LiVal + AAP	Lith → LiVal
16	X	O	O	X	X	X	LiVal + Lamo	Val → LiVal
17	X	X	X	X	O	O	LiVal + AAP	Lith → Val → LiVal

Lith, lithium; Val, valproate; AAP, any atypical antipsychotic medication; LiVal, lithium and valproate combination; Lamo, lamotrigine

^a^The order in which lithium and valproate were administered

^1^Lithium monotherapy

^2^VALPROATE monotherapy

^3^Lamotrigine with or without lithium, valproate, and antipsychotics

^4^Atypical antipsychotics monotherapy

^5^Lithium with atypical antipsychotics

^6^Valproate with atypical antipsychotics

### Clinical correlates of treatment response for each drug

Table 4 and Additional file 1: Table S2 display the results of a simple regression analysis of total Alda score. In lithium users, older age at onset and (hypo) manic episode at onset showed significant positive associations with total Alda score. The presence of mixed episodes and comorbid anxiety disorders or obsessive–compulsive disorder showed a significant negative association with the total Alda score in valproate users (Table 4).

**Table 4:** Clinical correlates of long-term treatment response in bipolar disorder patients: Results of simple regression analyses using the total Alda score as a dependent variable

Demographic and illness characteristics	Lithium (n = 29)^1^			Valproate (n = 56)^2^		
	Standardized beta	T	P	Standardized beta	T	P
Sex (female gender)	0.230	1.226	0.231	− 0.161	− 1.202	0.235
Age (current)	0.267	1.440	0.161	0.161	1.199	0.236
Education	− 0.138	− 0.712	0.483	0.142	1.056	0.296
Diagnosis (bipolar II disorder)	0.175	0.921	0.365	0.043	0.318	0.752
Age at onset	0.325	2.279	0.028	0.114	0.844	0.403
(Hypo)manic episode at onset	0.419	2.400	0.024	− 0.068	− 0.498	0.621
Pre-index-medication illness duration^a^	0.296	1.613	0.118	0.089	0.647	0.520
Frequency of episodes (frequent)						
Total	0.177	0.937	0.357	0.018	0.132	0.896
Major depressive	0.224	1.196	0.242	0.113	0.834	0.408
(Hypo)manic	− 0.153	− 0.807	0.427	− 0.090	− 0.665	0.509
Family history of psychiatric disorders	0.107	0.559	0.581	0.145	1.078	0.286
Family history of mood disorders	0.201	1.068	0.295	0.080	0.591	0.557
Any anxiety disorder	− 0.209	− 1.113	0.276	-0.475	− 3.967	< 0.001
Obsessive–compulsive disorder	-0.209	− 1.113	0.276	-0.393	− 3.145	0.003
Seasonality	− 0.017	− 0.072	0.944	0.221	1.377	0.177
Rapid cycling	0.338	1.865	0.073	− 0.049	− 0.362	0.719
Psychotic symptoms	− 0.363	− 2.022	0.053	− 0.068	− 0.498	0.621
Mixed episodes	− 0.163	− 0.859	0.398	− 0.289	− 2.196	0.033

^a^Duration from illness onset to start of the index medication

^1^Lithium monotherapy group

^2^Valproate monotherapy group

Among symptoms of depressive or manic episodes, increased appetite during depressive episodes was significantly associated with total Alda score in valproate users (Additional file 1: Table S2).

## Discussion

In this study, we evaluated long-term response to maintenance treatment with specific mood stabilizers including subjects under mood stabilizer combination therapy. We observed that patients who received combination therapy showed poorer treatment response compared to those who received monotherapy. We also identified distinct clinical correlates of response to lithium and valproate.

The initial Alda scale (Grof et al. 2002) stated that the systematic use of antidepressants, antipsychotics, or additional mood stabilizers should be given a score of 2 on item B5 in order to suggest that the link between improvement and a specific treatment is less certain. However, in ConLiGen projects, item B5 was modified to allow the systematic use of antidepressant or antipsychotic medication only (Schulze et al. 2010). Thus, subjects under mood stabilizer combination therapy were excluded from the beginning. Furthermore, a recent study by Scott and colleagues (Scott et al. 2019) even suggested that subjects under combination therapy with antidepressant/ antipsychotics (high B5 scorers) need to be excluded at first. However, combination treatment for BD is quite pervasive and rapidly increasing (Greil et al. 2012; Baek et al. 2014; Fung et al. 2019). If we were to exclude all patients under combination treatment, a limited number of patients could be included in subjects.

Of combination treatment, the mood stabilizer combination strategy occupies a unique position in the treatment of BD. Lithium and valproate are both first-line treatment agents used for maintenance treatment of BD. But the combination of two first-line treatment agents is generally recommended when a patient does not show a satisfactory response to a single first-line agent (Yatham et al. 2018). Thus, patients given mood stabilizer combination treatment are more likely to be poor treatment responders. If we exclude all patients under mood stabilizer combination treatment, we will be disregarding the characteristics of many poor treatment responders in the analysis of pharmacological and pharmacogenetic factors.

In line with the preceding discussion, the combination group showed worse long-term treatment response compared to both monotherapy groups (Fig. 1 and Table 2). The mood stabilizer combination group had the lowest mean Alda A score, indicating that their low treatment response is not derived from high B scores associated with combination treatment. No significant difference was observed between the combination group and the monotherapy groups (either the lithium or the valproate group) in terms of baseline illness characteristics indicating that the difference in long-term treatment response is not derived from baseline illness characteristics. A few studies explored long-term treatment responses of combination therapy in comparison to mood stabilizer monotherapy. The BALANCE study (Geddes et al. 2010) compared the long-term treatment responses of mood stabilizer monotherapy and combination therapy; they reported greater decrease of recurrences in combination therapy. In that study, patients were randomly allocated into monotherapy vs. combination therapy groups. By contrast, in our study, the combination of lithium and valproate was chosen for individual patients through their treatment processes. Therefore, most of our subjects in the combination group had experienced trials of various other treatment regimens before the current medications, indicating inadequate responses to previous treatments including lithium and/or valproate monotherapies (Table 3). In another study, Musetti et al. (Musetti et al. 2018) compared monotherapy and combination therapy groups in a naturalistic setting as in the current study. In that study, current regimens of the combination group were chosen after some other trials including mood stabilizer monotherapy. As a result, combination group had greater episode frequency previously, and the reduction rate of recurrence was higher due to worse previous illness course. So in some way, the findings from the study by Musetti et al. corroborates with our study findings showing that poor treatment responders were allocated into combination therapy group.

Systematic meta-analysis and post-hoc analysis of randomized controlled trials found no significant differences between lithium and valproate in terms of long-term efficacy (Cipriani et al. 2013; Kang et al. 2020). In contrast, observational studies (Garnham et al. 2007; Kessing et al. 2018), a randomized open trial (Geddes et al. 2010) and a population-based cohort study (Hayes et al. 2016) reported the superiority of lithium as a monotherapy agent for maintenance treatment of BD. Our study did not find significant differences in terms of treatment response between lithium and valproate. It is also unknown whether there are distinct clinical correlates of treatment response depending on type of mood stabilizer. Considering that lithium and valproate have distinct neurobiological targets (Chiu et al. 2013), different clinical factors could contribute to the long-term response to each medication.

Prior studies on the clinical correlates of the long-term effects of mood stabilizers have more focused on lithium. A recent meta-analysis (Hui et al. 2019) reported that mania-depression-interval sequence (compared to depression-mania sequence), absence of rapid cycling, absence of psychotic symptoms, shorter pre-lithium illness duration, family history of bipolar disorder, and later illness onset were associated with better long-term treatment response to lithium. In our study, older age at onset and (hypo)manic episode at onset were significantly associated with long-term treatment response. Other factors that showed associations in the meta-analysis did not show significant associations in our study.

Regarding clinical correlates of valproate response, Gyulai et al. (Gyulai et al. 2003) reported that worse depression symptoms, rapid cycling, and comorbid alcohol use disorders were associated with the efficacy of valproate in a maintenance treatment. In addition, a study by Garnham et al. (Garnham et al. 2007) reported psychosis was associated with treatment response to valproate. In our study, comorbid anxiety disorder (Otto et al. 2006), obsessive–compulsive disorder (Shashidhara et al. 2015) and mixed episode (McIntyre et al. 2013; Young and Eberhard 2015), which are commonly known to be associated with poorer treatment responses, showed significant association with treatment response to valproate.

Several limitations of this study should be considered. First, the sample size is small. Subgroup analysis of BD-II patients could not be performed due to the limited sample size. For the same reason, evaluation of clinical correlates in the combination group was not possible. Second, this study measured treatment response retrospectively. However, as previously stated, retrospective evaluation is inevitable when evaluating long-term treatment response. We tried to carry out a comprehensive evaluation using diverse sources of information when determining long-term treatment response. Third, each patient’s treatment strategy can be affected their treating physicians’ preference. The clinicians’ preference can indirectly impact on clinical correlates of the treatment responses of each drug. Finally, the participants were recruited from a single tertiary academic teaching hospital, it may be difficult to generalize the study’s results.

Notwithstanding these limitations, this study has some strengths. As a naturalistic observational study, it may reflect the situation in real-world clinical practice. In addition, baseline disease characteristics were independently assessed in previous studies by the same authors before assessment of treatment response. Finally, even though the assessment of treatment response was retrospective, it was reliable, as the subjects were followed up for more than two years at a single institution.

## Conclusions

In this study, we took a novel approach to evaluating long-term response to mood stabilizers. By applying the Alda scale with minor modifications, we measured the responses to lithium and valproate including subjects under combination therapy. The combination treatment group showed poor treatment outcomes. We identified several factors related to the long-term treatment response to each drug. Further efforts are warranted towards the development of measurement methods and study designs that can handle the effects of combination treatment in clinical and genetic studies of BD.

## Supplementary information

**Additional file 1: Table S1.** Correlation between the evaluation tools of long-term treatment response. **Table S2.** Symptom profiles and long-term treatment response in bipolar disorder patients: Results of simple regression analyses using the total Alda score as a dependent variable.

## Data Availability

Not applicable.

## Abbreviations

BD-I: Bipolar I disorder
BD-II: Bipolar II disorder
BD: Bipolar disorder
ConLiGen: Consortium on Lithium Genetics
SCID: Structured Clinical Interview for DSM-IV Axis I Disorders
SPAQ: Seasonal Pattern Assessment Questionnaire
CGI: Clinical Global Impressions
CGI-BP-III-O: Item III of the Clinical Global Impressions scale for use in bipolar illness, overall
Alda A: A score of the Alda scale
Alda C: Total score of the Alda scale
SD: Standard deviation
FE: Fisher’s exact test
ANOVA: Analysis of variance
