# Supplementary material for: Defining phenotypes of long-term lithium and valproate response, including combination therapy: a modified application of the Alda scale in patients with bipolar disorders
Source: Int J Bipolar Disord. 2020 Nov 20;8:36. doi: 10.1186/s40345-020-00199-w (PMC7677416; doi:10.1186/s40345-020-00199-w)
Supplement: Supplementary file 1 — Additional file 1: Table S1. Correlation between the evaluation tools of long-term treatment response. Table S2. Symptom profiles and long-term treatment response in bipolar disorder patients: Results of simple regression analyses using the total Alda score as a dependent variable. [file 40345_2020_199_MOESM1_ESM.docx]

**Table S1. Correlation between the evaluation tools of long-term treatment response**

|  | Alda A | Alda total score | CGI-BP-III-O |
| --- | --- | --- | --- |
| Alda A | - |  |  |
| Alda total score | 0.833** | - |  |
| CGI-BP-III-O | -0.664** | -0.548** | - |

*Alda A*, A score of the Alda scale (Grof et al. 2002), *CGI-BP-III-O*, item III of the Clinical Global Impressions scale for use in bipolar illness, overall (Spearing et al. 1997)

** Correlation is significant at p$<$0.01

**Table S2. Symptom profiles and long-term treatment response in bipolar disorder patients: Results of simple regression analyses using the total Alda score as a dependent variable**

| Symptoms of mood episodes | Lithium users (n=29) | | |  | Valproate users (n=56) | | |  |
| --- | --- | --- | --- | --- | --- | --- | --- | --- |
|  | Standardized  beta | *T* | *P* |  | Standardized  beta | *T* | *P* |  |
| (Hypo)manic episodes | | | | | | | | |
| Elevated mood | 0.192 | 1.017 | 0.318 |  | 0.125 | 0.928 | 0.357 |  |
| Irritability | -0.222 | -1.184 | 0.247 |  | 0.072 | 0.528 | 0.600 |  |
| Grandiosity | -0.141 | -0.739 | 0.467 |  | 0.014 | 0.102 | 0.919 |  |
| Decreased sleep need | -0.075 | -0.391 | 0.699 |  | 0.195 | 1.462 | 0.149 |  |
| Talkativeness | 0.043 | 0.224 | 0.824 |  | -0.057 | -0.422 | 0.675 |  |
| Flight of idea | -0.224 | -1.196 | 0.242 |  | -0.007 | -0.048 | 0.962 |  |
| Distractibility | 0.219 | 1.166 | 0.254 |  | -0.176 | -1.310 | 0.196 |  |
| Hyperactivity | -0.075 | -0.391 | 0.699 |  | <0.001 | <0.001 | 1.000 |  |
| Excessive involvement in activity | 0.071 | 0.369 | 0.715 |  | 0.079 | 0.580 | 0.565 |  |
| Delusion | -0.233 | -1.246 | 0.224 |  | -0.186 | -1.392 | 0.170 |  |
| Hallucination | -0.153 | -0.807 | 0.427 |  | 0.074 | 0.538 | 0.593 |  |
| Depressive episodes | | | | | | | | |
| Depressed mood | 0.091 | 0.475 | 0.638 |  | 0.050 | 0.369 | 0.713 |  |
| Decreased interest | 0.117 | 0.610 | 0.547 |  | <0.001 | <0.001 | 1.000 |  |
| Appetite change |  |  |  |  |  |  |  |  |
| No appetite change | -0.126 | -0.660 | 0.515 |  | -0.071 | -0.525 | 0.601 |  |
| Decreased appetite | 0.231 | 1.236 | 0.227 |  | 0.208 | 1.565 | 0.123 |  |
| Increased appetite | -0.043 | -0.221 | 0.826 |  | -0.264 | -2.013 | 0.049 |  |
| Insomnia | 0.082 | 0.428 | 0.672 |  | 0.074 | 0.542 | 0.590 |  |
| Hypersomnia | 0.303 | 1.654 | 0.110 |  | -0.039 | -0.289 | 0.774 |  |
| Agitation | -0.130 | -0.680 | 0.502 |  | -0.195 | -1.462 | 0.149 |  |
| Retardation | -0.010 | -0.054 | 0.957 |  | 0.109 | 0.805 | 0.424 |  |
| Fatigue or loss of energy | 0.006 | 0.033 | 0.974 |  | 0.159 | 1.187 | 0.241 |  |
| Guilty feeling or worthlessness | -0.080 | -0.419 | 0.679 |  | -0.078 | -0.572 | 0.570 |  |
| Indecisiveness (concentration) | 0.117 | 0.610 | 0.547 |  | -0.015 | -0.110 | 0.913 |  |
| Suicidal ideation | 0.002 | 0.011 | 0.992 |  | 0.149 | 1.110 | 0.272 |  |
| Delusion | -0.030 | -0.155 | 0.878 |  | -0.055 | -0.405 | 0.687 |  |
| Hallucination | -0.029 | -0.149 | 0.883 |  | -0.112 | -0.825 | 0.413 |  |
